# Comparison of dispatching after motor vehicle accidents – effects of the TPS-eCall system on dispatching time

**DOI:** 10.1186/s12873-025-01361-2

**Published:** 2025-09-23

**Authors:** Bastian Brune, Fabian Haut, Maximilian Wolf, André Nohl, Frank Herbstreit, Christian Waydhas, Marcel Dudda, Lars Becker

**Affiliations:** 1 Department of Trauma, Hand and Reconstructive Surgery, University Hospital Essen, University Duisburg-Essen, Essen, Germany; 2 Directorate of Emergency Medical Services, Fire Department Essen, Essen, Germany; 3 Zentrum für Notfallmedizin, BG-Klinikum Duisburg, Duisburg, Germany; 4 Directorate of Emergency Medical Services, Fire Department, Oberhausen, Germany; 5 Department of Anesthesiology and Intensive Care Medicine, University Hospital Essen, University Duisburg- Essen, Essen, Germany; 6 Department of Orthopedics and Trauma Surgery, BG Klinikum Duisburg, Duisburg, Germany

**Keywords:** Emergency call, eCall, AACN, TraumaRegister DGU®, Public-safety answering point, PSAP, Dispatching, ISS, Golden hour of shock

## Abstract

**Background/Objectives:**

Over the past 50 years, the concept of the golden hour of shock was established as one of the central tenets of emergency trauma medicine. A shorter duration of prehospital care correlates with a positive change in outcome in numerous studies. Dispatching by the public safety answering points has hardly been discussed to date. Thanks to improved vehicle safety, additional accident data is now available to the emergency call centers.

**Methods:**

We investigated the effects of third-party system emergency calls (TPS-eCalls), which have become mandatory in new passenger cars in the EU in 2018, on dispatching in the emergency medical services (EMS). For this purpose, we linked the data of a public-safety answering point (PSAP) and an EMS. All emergency service deployments from 01/01/2023 to 31/12/2023 were evaluated. *N* = 1546 rescue missions were dispatched after motor vehicle accidents (MVA), 111 after TPS-eCall-alerts, 1435 after conventional alerts.

**Results:**

Dispatching in the PSAP currently took longer after TPS-eCall alerts than conventional alerts (01:39 ± 01:40 min vs. 02:41 ± 02:01 min, *p* ≤ 0.001). The differences were only significant in the case of accidents involving ≤ 2 passengers.

**Conclusions:**

TPS-eCall data will be available increasingly. The future expansion data availability offers the opportunity to include objective accident data (airbag deployment, number of occupants, change of velocity) in the dispatching process. Adequate technical connection can improve dispatching and shorten preclinical treatment, especially for complex events with more than 2 passengers.

## Introduction

### Medical treatment

The term “golden hour of shock” was first introduced by R Adams Cowley in 1975 [1]. Over the past 50 years, the concept of the golden hour of shock has been established as one of the central tenets of emergency trauma medicine. This principle has been repeatedly reassessed in modern contexts, with more recent studies from the last decade supporting the association between shorter prehospital time and improved outcomes [2–4]. These newer analyses complement the earlier foundational work from the 1980–2000s, highlighting that while the concept originated decades ago, its relevance continues to be confirmed in contemporary trauma systems. The idea of treating the right patient with the right equipment in the appropriate trauma center is still the basis of organized trauma treatment and certification processes worldwide. Less known than the “golden hour of shock” is the military concept of prehospital emergency medicine, in which the on-scene time of EMS for seriously injured patients should be shorter than “10 platinum minutes” [5]. Both concepts, along with multiple contemporary studies, support the idea of shortening prehospital time to improve outcomes [2–4, 6–12]. Time delays due to rural accident locations, longer distances to specialized hospitals and tactical limitatations remain challenges even in modern trauma systems.

While preclinical and especially clinical treatments are discussed in the current literature, dispatching by public safety answering points (PSAP) is rarely considered. Although shorter prehospital times can improve outcomes, literature indicates that dispatch time alone is not directly associated with outcome; rather, it is one of multiple factors influencing the overall prehospital interval. However, dispatching marks the beginning of the dogmatically trained “golden hour of shock” and the “10 platinum minutes” and shall be discussed as the first possibility to reduce prehospital time. Due to the availability of accident data automatically transmitted via emergency calls (eCalls) motor vehicle accidents (MVA) must be distinguished from other medical emergencies. Unlike other operations, the number of injured persons is unclear in this case.

### eCall-data

European Regulation 2015/758 regulates that new cars must be equipped with an automatic emergency call notification system. Emergency call systems (eCall systems) have been mandatory for new passenger cars and light commercial vehicles in Germany since 04/01/2018. The general requirements are shown in Table 1.

**Table 1:** General requirements for operating an eCall system

GPS receiver for determining the position of the vehicle	
GSM antenna for sending the emergency call	
Control unit for reporting the location	
Crash sensor for detecting the type of accident	
Hands-free system	
Separate emergency power supply	
Button for manually triggering the emergency call	
Indicator light	

In Germany, the automotive industry can choose between two different systems to contact a PSAP. Accident data can be transmitted via emergency call number 112 (112-eCall) or so-called third-party service eCalls (TPS-eCalls) to provide vehicle occupants with early assistance in medical emergencies. The main difference is that 112 eCalls establish a direct connection between the vehicle occupants and the emergency call center. TPS-eCalls, on the other hand, are routed through a service center. In both systems, eCalls can be triggered manually or automatically. Both systems contain a minimum set of data (MSD). The MSD includes among other data about the exact place and time of an accident. The contents of the MSD are shown in Table 2. Another very important difference between the eCall-systems are extended data sets (ESD), that are only in some TPS-eCall data sets.

**Table 2:** Parameters of the minimal set of data (= MSD)

ECall activation	Automatic or manual activation
Test-Call	Test use or real use
Plausible position information	Yes/ no
Vehicle type	Type of vehicle
VIN	Vehicle identification number
Drive type	Petrol, diesel, hybrid, electric drive
Time information	Time of activation
Position Latitude	Location at the time of activation (Latitude)
Position Longitude	Location at the time of activation (Longitude)
Direction of travel	Last direction of travel

In the local EMS system studied, emergency calls via 112 are answered by trained PSAP dispatchers who use a structured priority dispatch protocol. TPS-eCalls reach the PSAP via a dedicated non-priority line after being processed by the service center. The decision on the number and type of units dispatched (including ambulances and EPs) lies with the PSAP dispatcher and is determined by the information from his phone call with the occupants or the service center.

Due to increasing safety demands in the European New Car Assessment Program (Euro NCAP), further data availability is expected in ESDs. Possible extensions include objective information on the number of passengers, airbag deployments and velocity changes. Furthermore, an expansion to other vehicle systems (e.g., trucks and motorcycles) is planned.

### Potential benefits for PSAP, EMS and patients

While the potential benefits are anticipated, robust evidence demonstrating a definitive improvement in patient outcomes through TPS-eCall implementation is not yet available. This study focuses on dispatching process parameters, and future outcome-based research is required to confirm these benefits.

The development of standardized, evidence-based protocols for dispatching in PSAPs and for the effective use of telemetry data from MVA has long been discussed. ECall systems and their implementation in the rescue chain represent a way of forwarding objective accident data automatically from the vehicle manufacturer to the emergency medical service (EMS) personnel within seconds.

In 2018, the year in which eCall systems became mandatory in new cars in Germany, 40 people per million inhabitants died from MVA. While the number of fatalities has not steadily declined recently, the mandatory installation of eCall systems is expected to increase the number of eCalls and thus also provide an opportunity to optimize treatment in individual cases. An increasing number of eCalls may lead to an acceleration of the flow of information after MVA and potentially lead to faster treatment of life-threatening prehospital injuries and potentially survivable deaths [13]. The transmission of objective crash data can also enable an evaluation of specific accident mechanisms, which can lead to individualization of treatment in emergency services and hospitals.

### Aim

The aim of our study was to evaluate the dispatching time of the PSAP after the MVA in the city of Essen, Germany, in 2023 (01/01/2023 to 12/31/2023). We examined the effects of dispatching rescue resources with and without TPS-eCall alerts on the operational process in the EMS. An individual risk assessment after MVA was made possible by linking to an extended TPS-eCall dataset. The current work focused on an evaluation of the handling of TPS-eCall accident datasets by the PSAP in the German public emergency service. In contrast to many other emergency services, a relatively high proportion of German EMS units are manned with emergency physicians (EPs).

## Materials and methods

We analyzed monocentric collected call logs from one PSAP and one EMS control center in a prospective observational study of routine data. One of the authors (BB) works in the medical directorate of the EMS of the City of Essen, Germany. In the context of professional supervision, an overall assessment of the treatment data from the PSAP and the EMS was mandatory. The study was reviewed and approved by the Ethics Committee of the Medical Faculty of the University of Duisburg-Essen (20-9161-BO).

All PSAP and EMS data were anonymized before analysis in compliance with applicable privacy regulations. No identifiable patient data were processed. Consent was therefore not required.

The protocols were linked in a multistage process. All EMS deployments after MVAs from 01/01/2023 to 31/12/2023 were evaluated. In the call logs, alerts were filtered on the basis of keywords that indicated an MVA. In addition to the callout keywords, a caller number evaluation (TPS-eCall service center vs. other callers) was provided and linked to the EMS database. All operations that contained no reference to MVA in the operational keywords were subsequently excluded.

Our data distinguished between rescue resources with and without an emergency physician (EP). The option of alerting an EP in addition to an ambulance made dispatching more difficult for the PSAP. The availability of several EPs in the Essen EMS offered the PSAP the opportunity to access the resource at a low threshold. Compared with international emergency services with fewer available EPs, the probability of overtriage shifted. To enable international comparability, only the arrival of the first vehicle, corresponding to the dispatching of an ambulance in the system without EPs, was considered when calculating the data. However, we later considered specifically for Germany whether an EP’s qualification was subsequently dispatched or whether an EP who was present had an effect on follow-up dispatching.

We also analyzed differences depending on the number of people injured in the accidents. We assumed a higher disposition complexity (DC) with a larger number of patients. This may have applied to missions with several severely injured persons who were required to be transported to a hospital in particular. In the evaluation of accident events, we categorized accidents on the basis of the number of patients. In this initial assessment, we defined the categories as follows: none or one patient (disposition complexity 1 (DC1), 2 patients (DC2), and ≥ 3 or more patients (DC3)).

The eCalls received by the PSAP in Essen, Germany, via 112-eCalls could not be distinguished from non-eCall alerts. This occurred because 112-eCalls cannot be recognized by the PSAP via their phone number. TPS-eCalls, on the other hand, can be identified on the basis of the defined number of callers of the service center. Thus, the TPS-eCalls were compared to a mixed group of non-eCalls and 112-eCalls.

Statistical analyses were conducted using appropriate tests based on data distribution. For normally distributed data, mean and standard deviation (SD) were reported and t-tests performed; for skewed distributions, non-parametric tests Mann-Whitney-U-tests were applied.

## Results

### Primary dispatching

From 01/01–12/31/2023, the PSAP of the Essen Fire Department dispatched 148,461 Emergency Medical Service (EMS) missions including 18,070 emergency physician missions, 53,371 ambulance missions and 1629 intensive care transports were considered as urgent missions, 75,391 as non-urgent patient transports. 1546 operations were triggered by MVAs. In 1435 cases, a conventional alarm was raised following information from parties involved in the accident. In 111 cases, a TPS-eCall Service Center contacted the PSAP by telephone. A total of 2409 rescue vehicles were involved in these 1546 deployments. TPS-eCall alerts accounted for 7.2% of all MVA-related EMS dispatches during the study period.

**Fig. 1:**
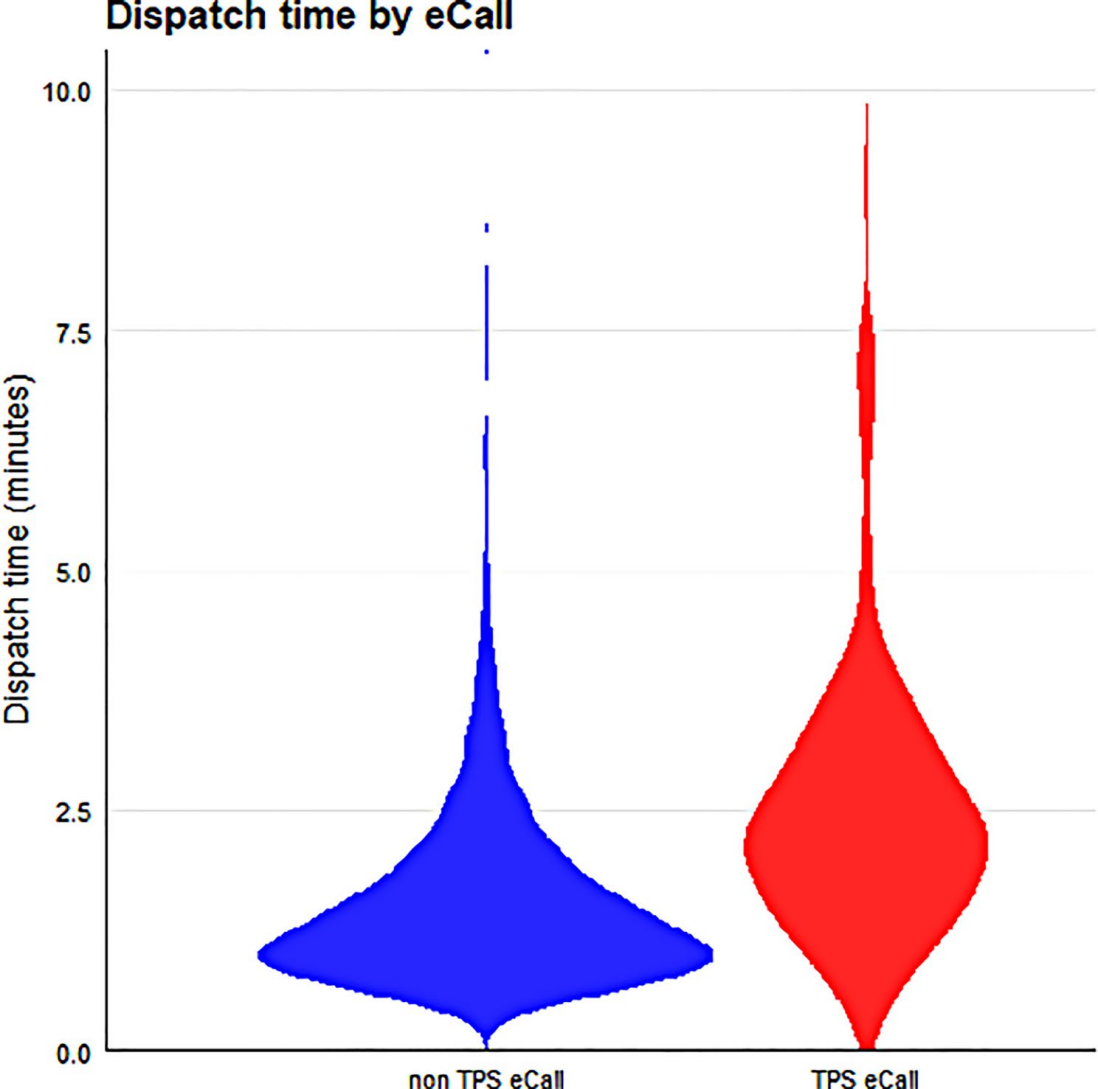
Average dispatching time ± standard deviation in minutes non-TPS-eCall vs. TPS-eCall

The duration of dispatch from call receipt in the PSAP to alerting the EMS unit was 01:44 ± 01:43 min for the entire collective. Dispatching was significantly shorter for interventions that were not initiated by TPS-eCall service centers (01:39 ± 01:40 vs. 02:41 ± 02:01 min, *p* ≤ 0.001) (Fig. 1). The involvement of EPs did not differ significantly between the categories, with 18% EP involvement in TPS-eCall dispositions and 22% non TPS-eCall dispositions.

### Follow-up dispatching

In the studied EMS system, ambulances are staffed at an Advanced Life Support (ALS) level. Follow-up dispatching (FUD) was interpreted as an qualitative or quantitative undertriage of the PSAP-dispatcher. A comparison of the TPS-eCall and non TPS-eCall categories revealed differences in the number of additional EMS units ordered (Table 3). There were significantly more additional requests for further emergency resources after TPS-eCall alerts (9% vs. 23.4%, *p* ≤ 0.001). Overtriage was not directly measured in this analysis.

**Table 3:** Numbers of follow-up dispatched (FUD) EMS units

	Follow-up dispatched EMS units			
	0	1 AMB	1 EP	≥ EMS unit
TPS-eCall (*n* = 111 operations)	85 (76.6%)	12 (10.8%)	4 3.6%)	10 (9%)
Non TPS-eCall (*n* = 1435 operations)	1310 (91.3%)	59 (4.1%)	41 (2.9%)	25 (1.7%)
Total (*n* = 1546 operations)	1395	71	45	35
	(90.2%)	(4.6%)	(2.9%)	(2.3%)

#### Follow-up dispatching depending on the qualifications of the EMS

In 1212 cases (*n* = 1125 (92.8%) non TPS-eCall vs. *n* = 87 (7.2%), TPS-eCall) there was no primary alerting of an EP. The subsequent alerting of an EP was necessary in 4 cases (3.6%) (TPS-eCall) and 41 cases (2.9%) (non TPS-eCall) (Table 4). The small absolute difference in incidence of secondary EP dispatches (3.6% vs. 2.9%) was not statistically significant and may not be clinically relevant given the sample size. In the event of a primary alert of an EP, emergency resources were subsequently alerted in 334 cases (310 (92.8%) non TPS-eCall vs. 24 (7.2%) TPS-eCall). After the primary alerts of an EP, there were no significant differences in the subsequent alerts.

**Table 4:** Follow-up dispatches with or without on-scene EP

	0	1 AMB	1 EP	≥ EMS unit
	**Follow-up dispatched EMS unit without on-scene EP**			
TPS-eCall (*n* = 87 operations)	64 (73.6%)	10 (11.5%)	4 (4.6%)	9 (10.3%)
Non TPS-eCall (*n* = 1125 operations)	1031 (91.6%)	36 (3.2%)	40 (3.6%)	18 (1.6%)
Total (*n* = 1212 operations)	1095	46	44	27
	(90.3%)	(3.8%)	(3.6%)	(2.2%)
	**Follow-up dispatched EMS unit with on-scene EP**			
TPS-eCall (*n* = 24 operations)	21 (87.5%)	2 (8.3%)	0 (0%)	1 (4.2%)
Non TPS-eCall (*n* = 310 operations)	279 (90%)	23 (7.4%)	1 (0.3%)	7 (2.3%)
Total (*n* = 334 operations)	300	25	1	8

### Dispatching complexity

**Fig. 2:**
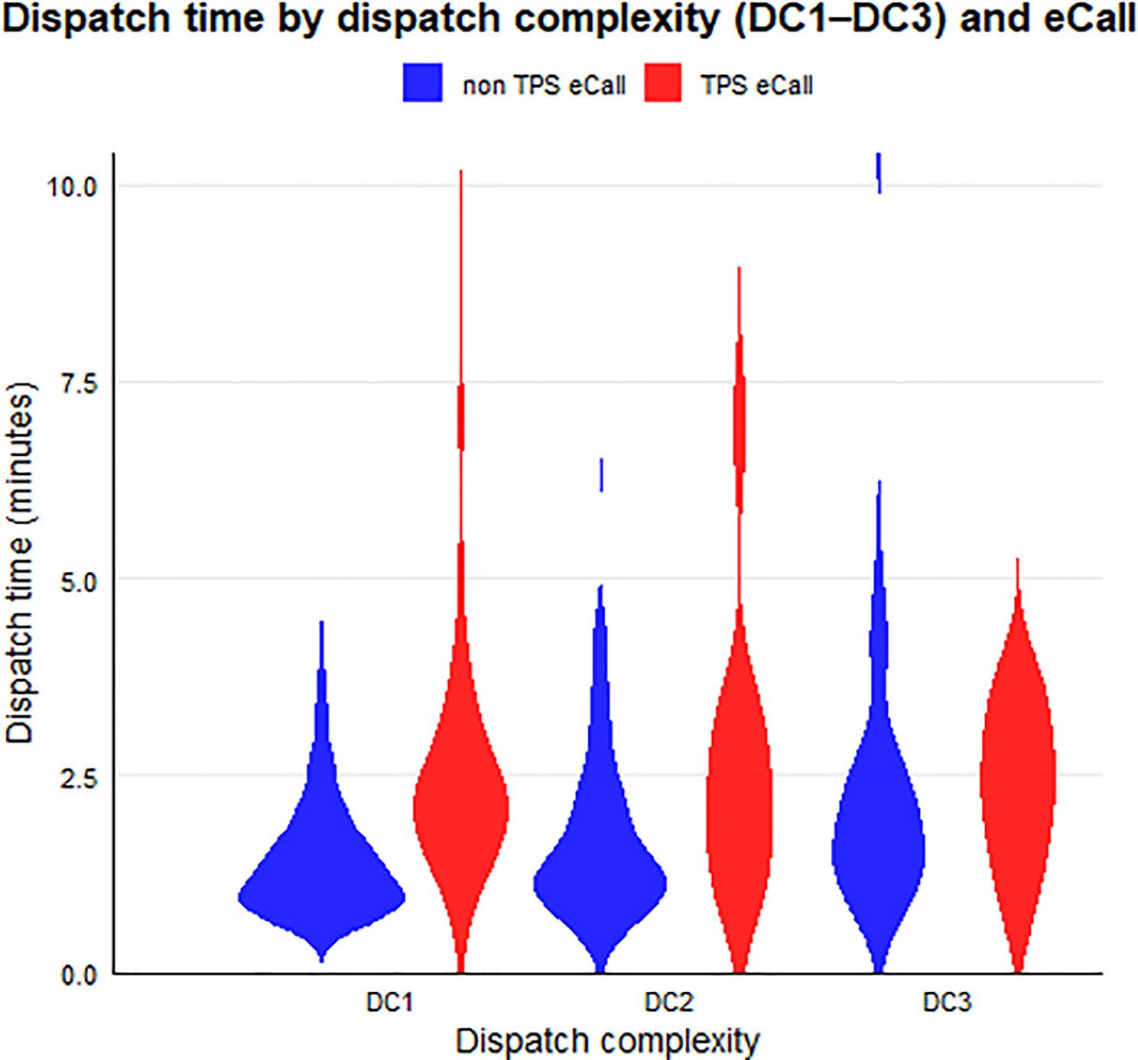
Average dispatching time ± standard deviation in minutes non-TPS-eCall vs. TPS-eCall depending on dispatching complexity

The duration of the dispatching process was significantly longer for the TPS-eCall interventions for DC1 and DC2 (Fig. 2). When differentiating by dispatching complexity, DC1 (TPS-eCall *n* = 19: 02:38 ± 01:44, non TPS-eCall *n* = 1278: 01:37 ± 01:41, *p* < 0.001) and DC2 (TPS-eCall *n* = 73: 02:46 ± 02:16, non TPS-eCall *n* = 105: 01:49 ± 01:26, *p* < 0.05) were significant. The differences in DC3 were not significant. There were no differences when the categories of eCall-associated accidents were compared. Non TPS-eCall-associated accidents were significantly different in all comparisons (DC1 vs. DC2: *p* < 0.05, DC2 vs. DC3 *p* < 0.05, DC1 vs. DC3 *p* < 0.001).

## Discussion

The common belief is that brief but precise EMS treatment with a short total prehospital time benefits patients with urgent treatment indications, as shown in 2 meta-analyses from 2015 to 2020 [2, 3]. Even if the concepts of the EMS and hospitals are increasingly considered in conjunction, the EMS is rarely the central focus of scientific considerations [3]. Documentation in the TraumaRegister of the German Trauma Society^®^ (TR-DGU) begins when the EMS is alerted. The prehospital time (“call-to-door”) is not divided into further time segments, such as the activation time, response time or the on-scene time of the EMS. Patients who died before arrival at the hospital are not included in the TR-DGU [14]. In the few studies in which the activation time was discussed, no change in outcome was found as a result of the activation time, neither for severe thoracic injuries nor for polytraumatized persons [15, 16]. However, the more frequently discussed preclinical overall treatment duration (“call-to door”) had an impact.

Emergency call times, dispatching intervals and standardization in the dispatching process can rarely be discussed in the context of accident-specific patient outcomes due to a lack of data linkages. Nevertheless, dispatchers throughout Europe initiate cost-intensive operations, mostly due to accident mechanisms, and decide on the necessity of EP treatment [17]. However, it should be noted that the assessment of the ideal treatment location (hospital selection) by the PSAP differs between the dispatching and the EMS during treatment [4, 18]. Both the PSAP and EMS could adjust their decisions on treatment urgency and destination hospitals with more objective accident data.

To illustrate the potential impact of better decisions, reference is given to the high number of potentially avoidable preclinical deaths [13]. Kleber et al. described a 15% proportion of (potentially) preventable, because treatable, causes in an analysis of trauma-related deaths in an urban EMS, which is comparable to the EMS in Essen, Germany. The small but significant difference in activation time could enable early treatment and thus improve outcomes in individual cases of potentially preventable deaths. These minor differences are insignificant compared to the total preclinical time. Their relevance is therefore more significant in treatable and treated injuries than in untreatable or untreated injuries. However, in cases where not enough or insufficiently qualified personnel have been assigned due to undertriage, resulting delays until treatment might be significantly longer than the short difference in dispatch time may occur. Even if the response time is met and additional EMS personnel are immediately requested, there will still be a significant delay until treatment can be provided by the additional EMS personnel. Another benefit arises with all eCalls when automatic triggering initiates dispatch processes after MVA that an unconscious driver would not have been able to initiate.

In a 2020 meta-analysis, Bedard et al. tried to substantiate the effects of prehospital time on the outcomes of trauma patients [3]. Unfortunately, the evaluation of prehospital treatment was based on in-hospital deaths with an observation period of 30 days, while prehospital deaths were excluded. In the current TR-DGU^®^ annual report from 2024, two time points with an increased rate of death were identified in patients: within the first 48 h after trauma and around the 7th day after the accident [14]. The annual report states 9836 severely injured patients after MVAs. With a national population of 83.5 million inhabitants the incidence is 119 per million inhabitants [14]. The questions of whether mortality is attributable to preclinical or clinical treatment strategies and whether the dispatching and the resulting reduction in preclinical time have an impact on mortality remain unanswered.

Our results reveal significant differences in the dispatching process, with shorter activation times for nonTPS-eCall activations. A shorter time may improve outcomes for potentially treatable causes of death. Due to the relatively low number of TPS-eCall cases, only 1 with high ISS and documentation in the TR-DGU^®^ and no patient with an immediate need for treatment due to airway obstruction, tension pneumothorax or critical hemorrhage was identified [19]. Lovely et al. described in 2018, again excluding prehospital deaths and admission time, that on-scene time and transport time are less relevant to outcome than the injury severity score (ISS) in MVA [20]. Even if ISS is the main determinant of outcome, early treatment may have a positive effect. This interpretation is also supported by Brown et al., who described a prolongation of hospitalization by a factor of 1.16 for each additional minute on-scene [21]. Although Brown et al. did not refer to the deployment time, a worsening of the findings at high ISS without adequate treatment seems to have a conclusive correlation regardless of the extension of the deployment time or the on-scene time. As already mentioned, EMS shortages lead to a longer extension than a short delay in activation time when dispatching is correct.

We also found significant differences between TPS-eCall and conventionally dispatched non TPS-eCall-operations with a lower or middle dispatching complexity (DC1 or DC2). In the case of complex MVA (DC3), the dispatching times differ no longer. While the dispatching time for conventional alarms increased with the number of passengers, the dispatching time for TPS-eCalls was constant. Unfortunately, the non TPS-eCall group is heterogeneous, including non-eCalls and 112-eCalls. This may over- or under-estimate differences, depending on the complexity.

In the detailed analysis, it should be noted that for all the results, conventional alarms and 112-eCalls are raised via emergency number 112. TPS-eCall alerts do not reach the emergency number 112 but instead address a non-prioritized telephone number in the PSAP. Due to purely telephone-based data transmission, it may be necessary to convert geodata into postal addresses when coordinating the location of an emergency. These technical disadvantages are possibly the reason for the significant differences in dispatching times. An identical connection to the emergency number 112 could lead to an improvement in alerts from TPS-eCall service centers.

Unfortunately, the evaluation of time differences is only possible after telephone contact with the PSAP. TPS-eCall data could be supplemented with the exact time of the accident event if the service centers were connected via a data interface with the PSAP. In the case of accidents without eCall data, the time of the accident will remain unknown, so this limitation persists in the comparison. Compared with conventional alerting without eCall data, a relative shortening of the dispatching process after TPS-eCalls via data interfaces is highly probable.

The explanation for the higher FUD rate, interpreted as a prior undertriage of the PSAP, may be caused by several factors. The reasons could be that the dispatchers in the PSAP are not yet trained in handling the available information or that dispatchers at the service centers are less in line with the guidelines when assessing accident incidents than the EMS personnel on site are.

Next, low case numbers can make interpretation more difficult for operations with higher dispatching complexity. However, with low case numbers but an identical percentage ratio of TPS-eCall alerts to non TPS-eCall-alerts, the number of FUD appears to be greater, particularly when no EP is alerted. During the study period, the S3 guidelines for the treatment of polytrauma/seriously injured patients were updated. It is therefore also possible that the EMS personnel still followed the previous guidelines, which provided a more generous indication for the activation of a trauma team and thus also an EP indication, while the EPs were already familiar with the new guidelines.

## Limitations

The current study is subject to several limitations. The number of eCall alerts and the number of complex dispositions are relatively low in the local study approach. The duration from the accident event to contact the PSAP is not traceable for either the TPS-eCall- or the non TPS-eCall-alerts, so that only the dispatching at the PSAP can be evaluated. Unfortunately, TPS-eCalls do not reach the PSAP via the prioritized emergency number, so there may be a time delay compared with other alarms.

The technical connection and transmission of TPS-eCall events as data records can lead to a significant acceleration of the dispatching process, especially compared with alerts by persons who have been injured, but this cannot yet be verified. As eCall systems become more widespread, the relevance of accident reporting systems increases. Due to the lack of data availability, the current study did not follow up on hospital treatment. By including hospital treatment, an improved evaluation of the outcome may be possible.

Even without patient outcome data, presentation of a complete prehospital timeline (call-to-door time) could add context. This was not possible in the present study due to data limitations, but will be considered in future work. On-scene time was not analyzed separately, as the control group included a heterogeneous mix of conventional alarms and 112-eCall-missions. Therefore, no meaningful comparison was possible. This will be addressed in future work after adapting data collection.

## Conclusions

In the future, standardized dispatching must be established and evaluated for conventional alerts and, because of data availability, even more for eCall-alerts. Dispatchers must be trained to process and interpret eCall data to maintain higher dispatching quality by using eCall data.

A basic prerequisite for the adequate use of eCall data is the establishment of a data interface between the PSAP and the TPS-eCall service center. To understand aspects of prehospital time and its impact on outcomes, an understanding of dispatching, dispatching complexity and the use of objective prehospital data is desirable.

The evaluation of objective accident mechanisms can provide an additional step in analyses of registry data.

## Data Availability

The data analyzed in the study can be obtained from the author in anonymized form upon reasoned request and with the permission of the data owner. The personal rights of the persons involved in the accidents must be preserved. Requests to access the datasets should be directed to BB.

## Abbreviation

DC: Dispatching complexity
eCall: Emergency call
EMS: Emergency medical service
EP: Emergency physician
ESD: Extended set of data
FUD: Follow-up dispatching
ISS: Injury severity score
MVA: Motor vehicle accidents
MSD: Minimal set of data
PSAP: Public-safety answering point
TPS-eCall: Third-Party-Service-emergency call
TR-DGU®: TraumaRegister of the German Trauma Society
